# Genome Sequence of a Novel Human Gammapapillomavirus Isolated from Skin

**DOI:** 10.1128/genomeA.00439-17

**Published:** 2017-06-08

**Authors:** Sankhadeep Dutta, Alexis Robitaille, Magali Olivier, Dana E. Rollison, Massimo Tommasino, Tarik Gheit

**Affiliations:** a Infections and Cancer Biology Group, International Agency for Research on Cancer, Lyon, France; b Molecular Mechanisms and Biomarkers Group, International Agency for Research on Cancer, Lyon, France; c Department of Cancer Epidemiology, Moffitt Cancer Center, Tampa, Florida, USA

## Abstract

A new human gammapapillomavirus (HPV_MTS2) genome was isolated and fully cloned from a skin swab. The L1 open reading frame of HPV_MTS2 was 79% and 80% identical to those of its closest relatives, HPV type 149 (species Gamma-7 of the genus *Gammapapillomavirus*) and HPV isolate Dysk2 (GenBank accession no. KX781281), respectively, thus qualifying it as a new HPV type.

## GENOME ANNOUNCEMENT

Human papillomaviruses (HPVs) belonging to the genus *Gammapapillomavirus* (gamma-HPVs) have traditionally been classified as cutaneotrophic (1). However, a growing body of evidence suggests a much broader tissue tropism with gamma-HPVs detected in mucocutaneous areas of the anogenital region, oral and nasal mucosa, and various cutaneous and genital lesions (2–9). With the identification of new gamma-HPV genomes, the genus *Gammapapillomavirus* has been growing rapidly in recent years and is currently divided into 27 species. Here, we report the complete genome sequence of a novel HPV type obtained from a skin swab of a healthy individual.

The complete genome of a new HPV type (HPV_MTS2; 7,319 bp) was obtained by amplifying DNA from a human forearm skin swab using multiply-primed rolling circle amplification (RCA) according to the manufacturer’s instructions (illustra TempliPhi 100 amplification kit, GE Healthcare, USA). The amplified product was digested with *EcoRI* and cloned into the pUC19 vector for sequencing using a primer-walking strategy (GATC-Biotech, Germany), which covered nucleotides 647 to 6489 (5,843 bp) of the viral genome. To obtain the missing part of the viral genome, long-range PCR was performed on the RCA product template using TaKaRa LA Taq DNA polymerase and HPV_MTS2-specific primers (forward: 5′ TCCGCTTCTGTTACAATATACCA 3′; reverse: 5′ GTTTAGAAGCAGATATTCTTGC 3′). The amplicon was cloned in the pCR-XL-TOPO vector using the TOPO-XL PCR cloning kit (Invitrogen, USA) and sequenced. The sequence of the whole viral genome was confirmed by a strategy that implies the use of a proofreading *Pfu* DNA polymerase (Agilent Technologies, USA).

An HPV genome is considered to be a novel type if it shares less than 90% sequence similarity to the closest papillomavirus type in the L1 open reading frame (ORF) (1). The L1 ORF of HPV_MTS2 demonstrated 79% nucleotide homology to its closest relative, HPV type 149, belonging to species Gamma-7, and the newly identified HPV isolate Dysk2 (GenBank accession no. KX781281). However, the overall nucleotide homology between HPV_MTS2 and HPV isolate Dysk2 was 80%, and the homology between HPV_MTS2 and HPV type 149 was 79%. Overall, the G+C content of HPV_MTS2 was 37.8%. The genome contains five early (E1, E2, E4, E6, and E7) and two late (L1 and L2) ORFs, but no E5 ORF, a genomic organization typical of other gamma-HPVs. The long control region between L1 and E6 is 512 bp long and contains the TATA box (TATAAA), one polyadenylation site (AATAAA) for L1 and L2 transcripts, and four consensus palindromic E2-binding sites (ACC-N_6_-GGT). Two conserved zinc-binding domains [CxxC(x)_29_CxxC] separated by 36 amino acids were identified in E6 and one in E7 (10).

The consensus motif for binding to the pRB and its related proteins was observed in E7; however, serine was substituted for cysteine, thus forming the LxSxE motif (10). Such a modified LxSxE motif is common among members of the genus *Gammapapillomavirus*. In the carboxy terminus of E1 we identified a GPPNTGKS motif to be the putative ATP-binding site. Moreover, the E1 protein contained a cyclin interaction RXL motif required for viral replication (11).

To conclude, the genetic characterization of HPV_MTS2 expands the species composition of gamma-HPVs.

### Accession number(s).

The complete genome sequence of HPV_MTS2 is available in GenBank under the accession number KY780961.
